# Comparison of ^68^Ga-FAPI and ^18^F-FDG PET/CT in the Evaluation of Patients With Newly Diagnosed Non-Small Cell Lung Cancer

**DOI:** 10.3389/fonc.2022.924223

**Published:** 2022-07-04

**Authors:** Junhao Wu, Hao Deng, Haoshu Zhong, Tao Wang, Zijuan Rao, Yingwei Wang, Yue Chen, Chunyin Zhang

**Affiliations:** ^1^ Department of Nuclear Medicine, The Affiliated Hospital of Southwest Medical University, Luzhou, China; ^2^ Nuclear Medicine and Molecular Imaging Key Laboratory of Sichuan Province, Luzhou, China; ^3^ Academician (Expert) Workstation of Sichuan Province, Luzhou, China; ^4^ Department of Hematology, Clinical Medicine, Affiliated Hospital of Southwest Medical University, Luzhou, China; ^5^ Stem Cell Laboratory, The Clinical Research Institute, Affiliated Hospital of Southwest Medical University, Luzhou, China; ^6^ Department of the General Surgery, The Affiliated Hospital of Southwest Medical University, Luzhou, China

**Keywords:** 68 Ga-FAPI, 18 F-FDG, non-small-cell lung cancer (NSCLC), fibroblast activation protein (FAP), metastases

## Abstract

**Purpose:**

Several studies have demonstrated that ^68^Ga-FAPI PET/CT shows high intratumoral tracer uptake and low normal tissue uptake, allowing for excellent visualization of cancer. The purpose of this study was to compare the ability of ^68^Ga-FAPI and ^18^F-FDG PET/CT for the evaluation of newly diagnosed NSCLC.

**Materials and Methods:**

A prospective analysis of 28 individuals with histopathologically newly confirmed NSCLC that underwent ^68^Ga-FAPI and ^18^F-FDG PET/CT was conducted. The performance of two imaging modalities was compared based upon visual assessment, rates of cancer detection, and semi-quantitative parameters (target-to-background ratio [TBR], maximum standard uptake value [SUVmax]) for both primary tumors and metastases.

**Results:**

In total, this study enrolled 28 participants (13 male, 15 female; median age: 60.5 years, range: 34 – 78 years. <u>For primary tumors, ^68^Ga-FAPI and ^18^F-FDG PET/CT have similar detection performance (28 vs. 27). However, ^68^Ga-FAPI PET/CT was found to more effectively evaluate most metastases as compared to ^18^F-FDG PET/CT. ^68^Ga-FAPI PET/CT detecting more metastases present within the lymph nodes (53 vs. 49), pleura (8 vs. 7), liver (4 vs. 1), and bone (41 vs. 35).</u> The SUVmax and TBR values for ^68^Ga-FAPI were substantially superior to those for ^18^F-FDG in lymph node, pleural, and bone metastases. While the SUVmax for these two imaging approaches was comparable for hepatic metastases, ^68^Ga-FAPI exhibited a significantly higher TBR in relation to that of ^18^F-FDG. In addition, ^68^Ga-FAPI PET/CT demonstrates excellent N (80% [8/10]) and M (92.9% [26/28]) staging accuracy in NSCLC patients.

**Conclusions:**

^68^Ga-FAPI PET/CT as an examination modality is excellent for evaluation of newly diagnosed NSCLC. ^68^Ga-FAPI PET/CT improves the detection rates of most metastases and facilitating the superior staging of patients with newly diagnosed NSCLC, relative to that achieved by ^18^F-FDG PET/CT.

## Introduction

Cancer is one of the fundamental threats to human health and well-being, with lung cancer in particular remaining among the most common and deadliest tumors (1). Lung cancer is a heterogeneous classification of epithelial malignancy with a range of pathological and clinical manifestations. Broadly speaking, lung cancer cases are subdivided into non-small-cell lung cancer (NSCLC) and small cell lung cancer (SCLC) (2–4). For individuals with stage I – IIIA NSCLC, surgical resection is the optimal therapeutic intervention, but just 20-25% of patients are suited to undergo curative surgical resection (2, 3, 5, 6). The eligibility of newly diagnosed patients for such treatment is generally dependent on the degree of tumor involvement such that accurate tumor staging is essential and can affect both the prognostic evaluation and treatment of patients (5, 7). 18F-fluorodeoxyglucose (18F-FDG) positron emission tomography/computed tomography (PET/CT) imaging has emerged as the most widely used modality for diagnosing and systemically staging NSCLC. However, the utility of this approach can be limited by insufficient soft-tissue contrast and by elevated levels of physiological background activity in specific organs (1, 8, 9). Cancer-associated fibroblasts (CAF) are commonly linked to a poor cancer patient prognosis (10–16). CAFs frequently express elevated levels of the type II transmembrane serine protease fibroblast-activated protein (FAP) (17–19), which plays key roles in migratory, invasive, and angiogenic activity in oncogenic contexts (20–24). Recently, novel quinoline FAP-specific inhibitor-based PET tracers have been developed that can be used to precisely target fibrotic and tumor-associated stromal tissue (19, 25, 26). 68Ga-FAPI PET/CT exhibits a high degree of intratumoral tracer uptake, low normal tissue uptake, and rapid clearance, thus resulting in excellent tumor visibility and a great target to background ratio (26–29). In multiple recent research (18, 19, 25, 27, 28, 30–34), 68Ga-FAPI PET/CT was demonstrated to aid in the visualization of a diversity of tumors in addition to offering clear advantages as compared to 18F-FDG PET/CT when discerning lymph node, pleural, brain, and bone metastases.

Current research advances suggest that 68Ga-FAPI may be a more accurate and convenient alternative to 18F-FDG PET/CT for the diagnosis and staging of lung cancer. Therefore, this study was conducted to examine the performance of 68Ga-FAPI and 18F-FDG PET/CT for the evaluation of newly diagnosed NSCLC.

## Materials and Methods

### Patients

The Ethics Committee of Southwest Medical University Hospital approved the present study, which was conducted from July 2020 - October 2021 (Ethics committee approval No.: 2020035), and all patients signed a written informed consent form. The inclusion criteria for this study were as follows: (1) individuals ≥ 18 years of age; (2) individuals newly diagnosed with NSCLC that had not undergone any previous antitumor treatment; (3) individuals who underwent both ^68^Ga-FAPI and ^18^F-FDG PET/CT at a 1-week interval. Contributors were excluded if they: (1) underwent < 3 months of follow-up; (2) had undergone antitumor treatment prior to PET/CT imaging; or (3) harbored any other non-NSCLC primary tumors.

### PET/CT Imaging

Contributors were asked to fast, not received intravenous glucose, and avoid strenuous activity or prolonged exercise for a minimum of 6 h before intravenous ^18^F-FDG (3.7 MBq/kg) infusion, and patients also needed to have normal blood glucose levels. ^68^Ga-FAPI injection (1.85–2.59 MBq/kg) did not necessitate any specific fasting or glycemic preparation. A hybrid PET/CT scanner (uMI780, United Imaging Healthcare, Shanghai, China) was used to conduct all PET/CT imaging ~1 h following radiotracer administration. With the contributor’s arms raised above their head, an initial spiral CT scan was conducted from the top of the skull to the upper portion of the mid-thigh (current 120 mA; tube voltage 120 kV; matrix 512 × 512 pixels; slice thickness 3.00 mm; window width 300–500 HU; window level 40–60 HU). PET scanning was subsequently conducted using the same bed position utilized for CT scanning, with 1.5 min/position in 3D acquisition mode and 5–6 bed positions. The resultant outcomes were transferred to a post-processing workstation (v R002, uWS-MI, United Imaging Healthcare, Shanghai, China). PET attenuation correction was performed using CT data, with PET data reconstruction being conducted based upon an ordered subset estimation maximization algorithm (20 subsets, 2 iterations). The overall condition of each case, such as their body temperature, heart rate, blood pressure, and mental status, was assessed by a nuclear medicine physician within 2 h following injection.

### Image Review

Two experienced nuclear medicine physicians independently conducted visual, qualitative, and semi-quantitative interpretation of all ^18^F-FDG and ^68^Ga-FAPI PET/CT. Discrepancies were resolved through discussion and consensus. Patient PET/CT images were assessed in the coronal, axial, and sagittal planes. Positive lesions were identified by areas of non-physiological uptake above background in ^68^Ga-FAPI or ^18^F-FDG PET images. Positive lesions were combined with data from the corresponding CT scan images for further diagnosis, and their length were measured and recorded. Positive PET/CT lesions were further categorized as non-malignant lesions, primary tumors, distant metastases, or lymph node metastases. ^18^F-FDG PET/CT and ^68^Ga-FAPI imaging results were initially compared *via* a visual assessment in which the two images for each patient were assessed to establish their relative inferiority or superiority when detecting primary tumors (based upon tumor size and conspicuousness) and metastatic lesions (based upon numbers, involvement, and conspicuousness). Semi-quantitative analyses were then conducted by comparing ^18^F-FDG and ^68^Ga-FAPI radiotracer uptake within the same lesions. SUVmax was measured using the analytical workstation after the region of interest (ROI) surrounding the lesion had been defined by a physician. The TBR was defined as the difference in radiotracer uptake between the lesion and background, and was measured *via* dividing the SUVmax for a given lesion by the mean normalized uptake (SUVmean) for normal background tissue.

### Diagnostic Criteria

Histopathological findings were used for final diagnostic determinations for all primary tumors. When histopathological results were not available for metastases, final diagnosis was made based upon the results derived from multiple imaging modalities (MRI, enhanced CT, ultrasound, bone scan, PET/CT) and corresponding follow-up imaging. During follow-up, a suspicious lesion was considered to be malignant if it exhibited progressive growth or the number and/or size of suspect lesions declined following antitumor treatment.

### Statistical Analysis

Statistical evaluations were executed using SPSS (v 26.0; IBM, NY, USA). General data were compared through descriptive analyses, with categorical variables being listed as numbers with percentages, while continuous variables were listed as the mean ± SD. Chi-squared tests were used to compare numbers of positive lesions. Student’s t-tests were employed for comparing SUVmax and TBR values for specific lesions associated with ^68^Ga-FAPI and ^18^F-FDG PET/CT. Correlations between lesion length and metabolic parameters (TBR and SUVmax) were assessed through Spearman’s rank correlation analyses. A two-tailed P < 0.05 was the threshold of significance.

### Results

Generally, this study enrolled 28 cases (13 male, 15 female; median age: 60.5 years, range: 34-78 years). The basic features of these cases are detailed in **Table 1**.

**Table 1:** Basic patient characteristics.

NO.	Sex	Age	Pathology	Primary tumor site	Length (cm)	Metastases site	Staging
1	F	44	ADC	right upper lobe	5.9	LNM; LM; PM	IVA
2	M	61	ADC	left upper lobe	2.7	LNM; AM	IVA
3	M	66	ADC	right upper lobe	1.2	None	IA
4	F	46	ADC	left upper lobe	1.8	LNM; BM	IVB
5	F	48	SCC	left lower lobe	2.2	None	IA
6	F	57	ADC	left lower lobe	3.2	LNM; HM; BM	IVB
7	F	53	ADC	left upper lobe	1.1	None	IA
8	F	72	ADC	right upper lobe	1.2	None	IA
9	M	70	ADC	right upper lobe	2.3	None	IA
10	F	78	ADC	left lower lobe	8.1	LM; PM; BM	IVA
11	M	68	ADC	left upper lobe	2.1	LNM	IIIA
12	F	57	ADC	right middle lobe	2.8	LNM	IIIB
13	M	69	SCC	right lower lobe	3	None	IA
14	M	49	ADC	right upper/lower lobe	8.9	LNM, Pancreas, Kidney	IVB
15	F	46	ADC	right middle lobe	3.2	LNM	IIIB
16	M	63	ADC	left lower lobe	3.1	BM	IVB
17	F	68	ADC ADC	right lower lobe right middle lobe	1.9 1.8	LNM	IIIA
18	M	63	ADC	left upper lobe	1.2	LNM;HM; BM	IVB
19	M	71	SCC	right upper lobe	3.3	LNM; BM	IVA
20	M	67	SCC	right upper lobe	2.7	AM	IVA
21	M	34	ADC	right lower lobe	3.3	LNM; BM	IVB
22	F	58	ADC	right lower lobe	3.1	LNM; PM	IVA
23	F	61	ADC	left upper lobe	2.2	LNM	IIB
24	F	60	ADC	right upper lobe	3.4	None	IB
25	M	56	SCC	left lower lobe	4.3	LNM	IIIA
26	F	45	ADC	right middle lobe	1.9	None	IA
27	F	53	ADC	right upper lobe	2.3	None	IA
28	M	68	ADC	right upper lobe	2.5	None	IA

SCC, squamous cell carcinoma; ADC, adenocarcinoma; LNM, lymph node metastasis; LM, lung metastasis; PM, Pleural metastasis; AM, adrenal metastasis; BM, bone metastasis; HM, hepatic metastases.

The patients had been newly diagnosed with NSCLC, including 24 patients diagnosed with adenocarcinomas and 5 diagnosed with squamous cell carcinomas, with one patient (patient 17) having been simultaneously diagnosed with two primary tumors. In total, 16 patients underwent surgical resection, with 10 having simultaneously undergone mediastinal lymph node dissection. The remaining 12 patients underwent non-surgical antitumor treatment.

### Adverse Event

No patients developed any adverse events, discomfort, or abnormalities with respect to heart rate, body temperature, blood pressure, or mental status within 2 h following imaging agent injection.

### Comparison of Visual Assessment Outcomes

**Figure 1:**
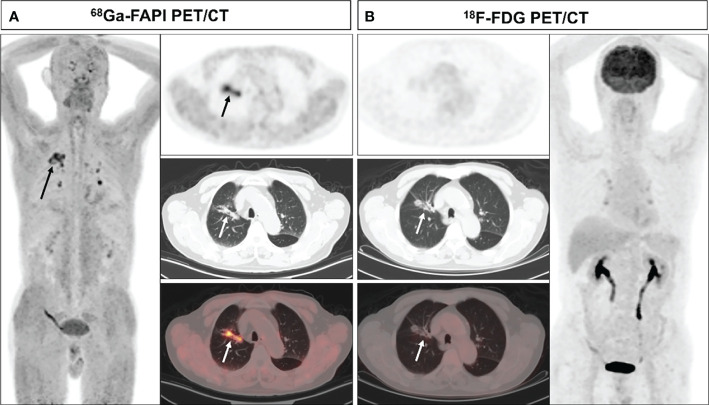
A 70-year-old male (patient 9) diagnosed with adenocarcinoma. ^68^Ga-FAPI PET/CT **(A)** revealed an adenocarcinoma lesion with increased FAPI uptake (solid arrows, SUVmax=6.3), while ^18^F-FDG PET/CT did not reveal any significant uptake in the primary lesion **(B)** solid arrows).

**Figure 2:**
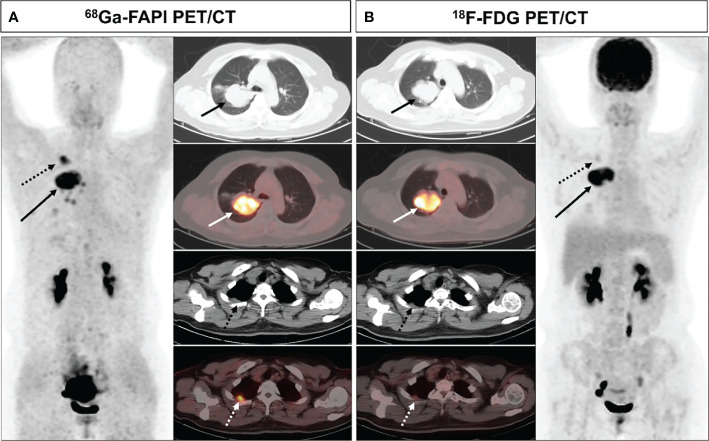
A 44-year-old female (patient 1) diagnosed with adenocarcinoma. ^68^Ga-FAPI PET/CT **(A)** revealed increased FAPI uptake in the primary lesion (solid arrows, SUVmax = 11.7) and pleural lesion (dashed arrows, SUVmax = 7.0). ^18^F-FDG PET/CT **(B)** also showed high FDG uptake in the primary lesion (solid arrows, SUVmax = 12.4), while the pleural lesion with only mild FDG uptake (dashed arrow, SUVmax = 2.2). The pleural lesion was deemed likely to be metastatic, as confirmed upon subsequent follow-up.

**Figure 3:**
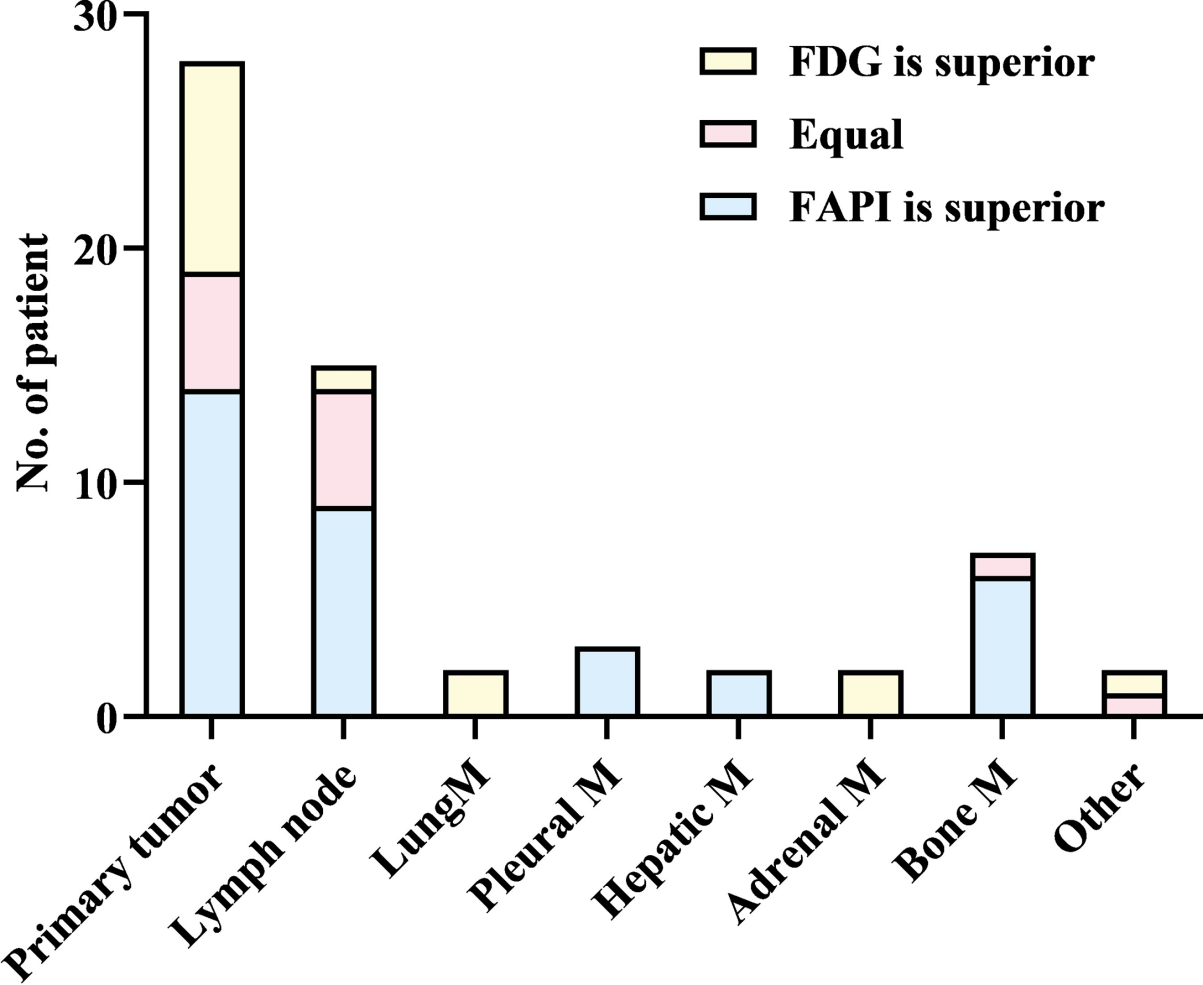
Visual assessment comparison for ^68^Ga-FAPI and ^18^F-FDG PET/CT. M = metastases.

Upon visual assessment, ^68^Ga-FAPI PET/CT enabled clearer metastatic and primary tumor visualization as compared to ^18^F-FDG PET/CT in a majority of patients. Specifically, ^68^Ga-FAPI outperformed ^18^F-FDG PET/CT for the visual evaluation of primary tumors (14/28 [50.0%] vs. 9/28 [32.1%]) (**Figure 1**), lymph node metastases (9/15 [60.0%] vs. 5/15 [33.3%]), pleural metastases (3/3 [100.0%] vs. 0/3 [0%]) (**Figure 2**), hepatic metastases (2/2 [100.0%] vs. 0/2 [0%]), and bone metastases (6/7 [85.7%] vs. 0/7 [0%]), but it performed less effectively for pulmonary (0/2 [0%] vs. 2/2 [100.0%]) and adrenal metastases (0/2 [0%] vs. 2/2 [100.0%]) (**Figure 3** **)**.

### Lesion Detection Analysis

**Figure 4:**
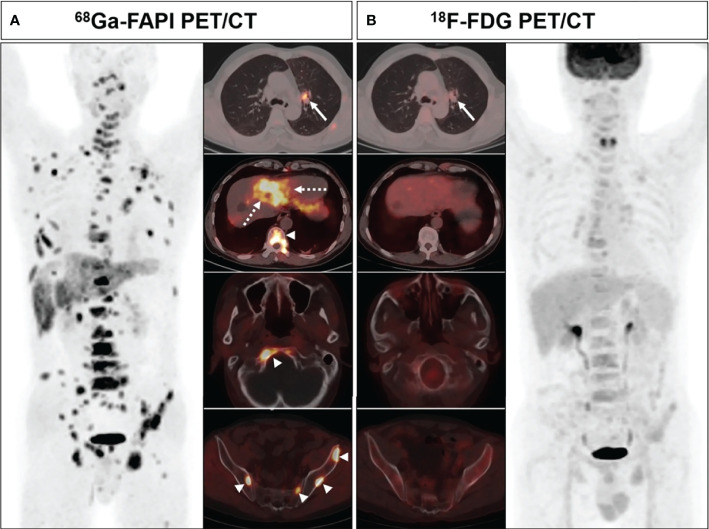
A 63-year-old male (patient 18) diagnosed with adenocarcinoma. ^68^Ga-FAPI PET/CT **(A)** showed intense tracer uptake in the primary tumor (solid arrows, SUVmax=10.0), liver metastasis (dashed arrows, SUVmax=7.6) and bone metastases (arrows, SUVmax=8.3-8.5). ^18^F-FDG PET/CT **(B)** showed primary lesion with mild FDG uptake (solid arrows, SUVmax=3.6), while no significant FDG uptake was showed in liver metastasis and multiple bone metastases.

^68^Ga-FAPI PET/CT outperformed ^18^F-FDG PET/CT in a lesion-based analysis when detecting hepatic (100% [4/4] vs. 25% [1/4]) and bone metastases (97.6% [41/42] vs. 83.3% [35/42]) (**Figure 4**), whereas ^68^Ga-FAPI was inferior to ^18^F-FDG PET/CT when utilized to detect adrenal metastases (0% [0/2] vs. 100% [2/2]). ^68^Ga-FAPI and ^18^F-FDG PET/CT performed similarly when used to detect primary tumors (96.6% [28/29] vs. 93.1% [27/29]), as well as lymph node (93.0% [53/57] vs. 86.0% [49/57]), pulmonary (100% [3/3] vs. 100% [3/3]), and pleural metastases (100% [8/8] vs. 87.5% [7/8]) (**Table 2**).

**Table 2:** Comparison of ^68^Ga-FAPI and ^18^F-FDG PET/CT semi-quantitative imaging parameters.

Parameter	Imaging method	Primary tumor	Lymph node metastasis	Lung metastasis	Pleural metastasis	Hepatic metastasis	Adrenal metastasis	Bone metastasis
Number of lesions		29	57	3	8	4	2	42
Positive detection	^68^Ga-FAPI	28	53	3	8	4	0	41
	^18^F-FDG	27	49	3	7	1	2	35
	*P*	0.554	0.222	1.000	0.302	0.028	0.046	0.026
SUVmax	^68^Ga-FAPI	9.3 ± 4.6	8.4 ± 4.3	2.4 ± 1.6	10.8 ± 3.6	6.2 ± 2.1	1.2 ± 0.4	11.2 ± 5.5
	^18^F-FDG	9.9 ± 6.9	6.4 ± 4.7	2.9 ± 1.9	5.5 ± 3.0	3.4 ± 0.27	6.4 ± 3.3	6.5 ± 3.9
	*P*	0.631	0.003	0.192	<0.001	0.062	0.237	<0.001
TBR	^68^Ga-FAPI	26.3 ± 18.8	10.6 ± 6.3	3.4 ± 1.8	9.1 ± 2.8	11.4 ± 5.3	1.6 ± 0.1	16.2 ± 11.2
	^18^F-FDG	24.0 ± 21.6	6.1 ± 4.9	4.8 ± 3.2	6.2 ± 3.3	1.3 ± 0.3	3.3 ± 2.5	5.9 ± 5.8
	*P*	0.589	<0.001	0.215	0.001	0.027	0.500	<0.001

### Comparison of Different Pathological Types

Evaluation of metabolism of primary tumors and lymph node metastases based on pathological type. For primary lung adenocarcinoma, there was no statistically significant difference in SUVmax (9.4 ± 4.8 vs. 8.7 ± 6.2, P = 0.572) and TBR (26.5 ± 19.9 vs. 19.7 ± 18.6, P = 0.131) between ^68^Ga-FAPI and ^18^F-FDG PET/CT. For primary lung squamous cell carcinoma, there was also no statistically significant difference in SUVmax (9.0 ± 4.1 vs. 15.8 ± 8.0, P = 0.156) and TRB (25.5 ± 14.1 vs. 44.2 ± 25.6, P = 0.16) between the two examination. For lymph node metastasis, The SUVmax (8.4 ± 4.3 vs. 5.9 ± 4.2, P =0.001) and TBR (10.8 ± 6.4 vs. 5.7 ± 4.6, P = 0.001) of lymph node metastases from adenocarcinoma were significantly higher in ^68^Ga-FAPI than in ^18^F-FDG PET/CT. In contrast, SUVmax (9.0 ± 5.3 vs. 11.2 ± 6.9, P = 0.077) and TBR (9.1 ± 5.2 vs. 10.1 ± 6.3, P = 0.227) for lymph node metastases from squamous cell carcinoma were not statistically significantly different between the two examination modalities.

### Comparison of Semi-Quantitative Parameters

The SUVmax and TBR values for ^68^Ga-FAPI and ^18^F-FDG PET/CT did not differ significantly when used for detecting primary tumors, pulmonary metastases, and adrenal metastases, while the SUVmax and TBR of ^68^Ga-FAPI were substantially superior to those for ^18^F-FDG PET/CT when used to detect lymph node, pleural, and bone metastases. Although there was no significant difference in SUVmax between these two imaging modalities in detecting liver metastases (P = 0.062), ^68^Ga-FAPI had significantly greater TBR values relative to ^18^F-FDG (P = 0.027) (**Table 2**).

### The Relationship Between Lesion Length and Suvmax

**Figure 5:**
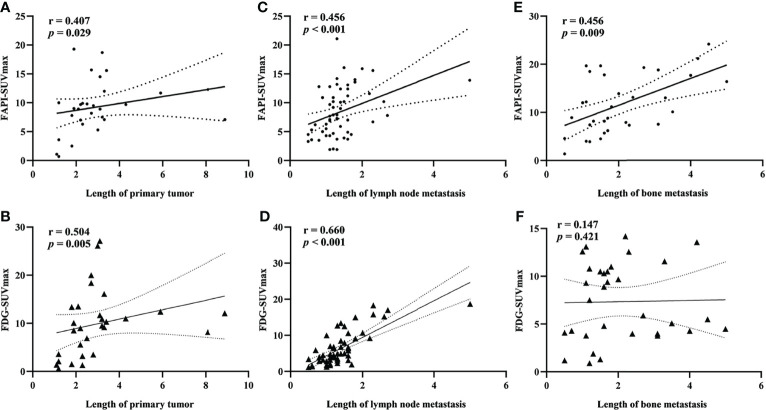
Spearman rank correlation analysis of the relationship between SUVmax value and lesion length for primary tumors **(A)**. FAPI-SUVmax; **(B)** FDG-SUVmax), lymph node metastases **(C)**. FAPI-SUVmax; **(D)** FDG-SUVmax), and bone metastases **(E)**. FAPI-SUVmax; **(F)** FDG-SUVmax).

Significant correlations between lesion length and FAPI-SUVmax were noted for primary tumors, lymph node metastases, and bone metastases in Spearman’s correlation analyses, while FDG-SUVmax values were only correlated with lesion length for primary tumors and lymph node metastases but not for bone metastases (**Figure 5**).

### N and M Staging

**Figure 6:**
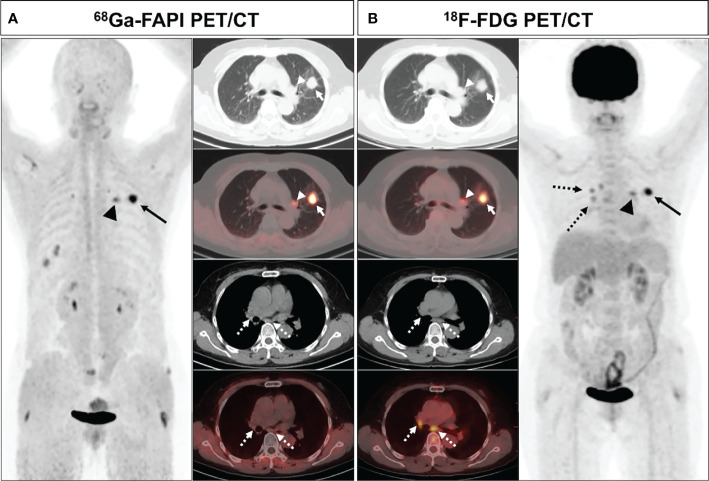
A 61-year-old female (patient 23) diagnosed with adenocarcinoma. ^68^Ga-FAPI PET/CT **(A)** revealed intense FAPI uptake in the primary tumor (solid arrows, SUVmax = 9.7) along with moderately increased uptake in the left pulmonary hilar lymph node (arrows, SUVmax = 5.0), whereas there was no FAPI uptake in the subcarinal and right pulmonary hilar lymph nodes (dashed arrows). ^18^F-FDG PET/CT **(B)** demonstrated intense FDG uptake in the primary tumor (solid arrows, SUVmax = 9.0) with moderate uptake in the left pulmonary hilar (arrows, SUVmax = 4.5), subcarinal, and right pulmonary hilar (dashed arrows, SUVmax = 3.5) lymph nodes. Pathological biopsy confirmed metastasis in the left hilar lymph node, while no metastasis was found in the subcardiac or right hilar lymph nodes.

Ten of all patients underwent mediastinal lymph node dissection. A total of 180 lymph nodes underwent pathological biopsy, of which 11 were malignant and 169 were benign. The sensitivity and specificity of ^68^Ga-FAPI and ^18^F-FDG PET/CT for detecting lymph node metastasis were 81.8% (9/11), 97.6% (165/169) and 72.7% (8/11), 88.8% (150/169), respectively (**Figure 6**). ^68^Ga-FAPI PET/CT led to a lower N-stage in 1 patient owing to overlooked lymph node metastases and a higher N-stage in 1 patient owing to the detection of additional false-positive lymph nodes. In contrast, ^18^F-FDG PET/CT detected additional false-positive lymph nodes in 5 patients resulting in higher N staging for these individuals. Overall, N-staging of NSCLC patients based on ^68^Ga-FAPI-FAPI results was more accurate than ^18^F-FDG PET/CT results in these same patients (80% [8/10] vs. 50% [5/10]), but there was no significant difference between the two values (p=0.16).

Distant metastatic lesions were found in 12 of all patients (42.9%). ^68^Ga-FAPI PET/CT failed to detect adrenal metastases in two patients, resulting in decreased M stage. ^18^F-FDG PET/CT resulted in incorrect M staging due to 1 false positive adrenal lesion and 2 false negatives for bone metastases. The overall M-staging accuracy of ^68^Ga-FAPI PET/CT was slightly higher than that of ^18^F-FDG PET/CT (92.9% [26/28] vs. 89.3% [25/28]), but the values were not statistically different between them (P=0.64).

## Discussion

The current exploration is to explore the comparative performance of ^68^Ga-FAPI and ^18^F-FDG PET/CT in the evaluation of patients with newly diagnosed NSCLC. Overall, these results show that ^68^Ga-FAPI PET/CT provides better lesion visualization and staging accuracy than ^18^F-FDG PET/CT in NSCLC.

One recent analysis (31) comparing ^68^Ga-FAPI and ^18^F-FDG reported no significant differences between these two technologies with respect to primary lung cancer detection rates or associated SUVmax or TBR values, in line with our findings. In contrast, Wang et al. (35) reported that ^68^Ga-FAPI yielded significantly higher SUVmax and TBR values as compared to ^18^F-FDG PET/CT, leading them to conclude that this former technology is better suited to the detection of early-stage lung cancer. AS their analysis specifically included individuals with large tumors (Mean size: 3.3 cm) and advanced disease, this may account for their inconsistent results. In addition, no significant differences in SUVmax and TBR were found in ^68^Ga-FAPI and ^18^F-FDG PET/CT for different pathological subtypes of primary tumors.

At present, surgical tumor resection is the benchmark of care for early-stage NSCLC patients. The capability of predicting and detecting regional lymph node metastases in these patients performs a central task in treatment planning and associated management efforts (36, 37). While ^18^F-FDG PET/CT imaging is frequently employed as a screening tool to stage lung cancer patients, it exhibits relatively low sensitivity for small metastatic lesions located within lymph nodes (8, 9). In contrast, we discovered that ^68^Ga-FAPI PET/CT was capable of detecting lymph node metastases more reliably than was ^18^F-FDG PET/CT, yielding higher SUVmax and TBR values for these metastases relative to the latter imaging modality. As ^68^Ga-FAPI PET/CT imaging can detect lymph node metastases at an earlier stage, it has the potential to increase occult lymph node metastasis detection, guiding the more accurate staging of NSCLC patients. However, for lymph node metastases from squamous cell carcinoma, SUVmax and TBR of ^68^Ga-FAPI were not significantly different compared to ^18^F-FDG. The ability of ^68^Ga-FAPI PET/CT to detect lymph node metastasis in squamous cell carcinoma still requires further and larger data studies. In patients undergoing mediastinal lymph node dissection, ^68^Ga-FAPI detected fewer mediastinal false-positive lymph nodes relative to ^18^F-FDG PET/CT, indicating that ^68^Ga-FAPI is more specific and has the potential to reduce the rate of unnecessary treatment in patients with NSCLC.

Our analyses additionally revealed ^68^Ga-FAPI to be superior to ^18^F-FDG PET/CT when used for the detection of hepatic, pleural, and bone metastases, in line with prior evidence (28, 35, 38, 39). This is ascribable to the reduced physiological uptake of the ^68^Ga-FAPI radiotracer and associated sensitivity gains. High levels of hepatic glucose metabolism have the potential to mask FDG uptake by metastatic lesions within this organ, while the use of ^68^Ga-FAPI PET/CT may enable the more reliable detection of these lesions. ^68^Ga-FAPI PET/CT is also capable of facilitating the early detection of occult bone and pleural metastases to guide more appropriate patient staging and treatment efforts. Unfortunately, we found discovered that ^68^Ga-FAPI PET/CT exhibited low sensitivity when used to detect adrenal metastases, suggesting that such lesions may be not associated with substantial fibrotic activity. However, ^18^F-FDG PET/CT is also not effective in diagnosing adrenal metastases due to its low specificity, suggesting that a combination of CT imaging and other modalities is necessary to ensure an accurate diagnosis. High physiological uptake in normal organs masks lesions, or metastases with low FDG uptake or small size are difficult to detect on ^18^FDG-PET/CT, which may lead to low detection rates on ^18^F-FDG PET/CT. The superiority of ^68^Ga-FAPI over ^18^F-FDG PET/CT for visual assessment and detection of most metastases may be attributed to the higher FAPI accumulation in the lesion and lower FAPI accumulation in normal organs.

There are multiple limitations to the present analysis. For one, the number of included contributors was relatively small, and the variety of NSCLC pathological types was limited, thus potentially contributing to some degree of bias in the overall study results. Second, accurate pathological results were not available for many suspicious metastatic lesions in individuals with advanced NSCLC as it is generally impractical and unethical to conduct biopsies of these samples. Third, the minimum follow-up duration for patients in this study was just 3 months, and future studies should thus utilize an extended follow-up interval.

## Conclusion

In summary, these results indicate that ^68^Ga-FAPI PET/CT imaging demonstrates desirable performances when used for the initial staging of newly diagnosed NSCLC. Moreover, ^68^Ga-FAPI exhibits significantly better diagnostic efficacy relative to that of ^18^F-FDG PET/CT imaging when used to detect metastatic lesions in the lymph nodes, pleura, liver, and bone. Therefore, ^68^Ga-FAPI PET/CT is expected to be a viable imaging modality for staging and management of patients with NSCLC, and may be an ideal alternative to ^18^F-FDG PET/CT.

## Data Availability Statement

The raw/processed data required to reproduce these findings cannot be shared at this time as the data also forms part of an ongoing study. Requests to access these datasets should be directed to the corresponding author.

## Ethics Statement

The Ethics Committee of Southwest Medical University Hospital approved the present study, which was conducted from July 2020 - October 2021 (Ethics committee approval No.: 2020035), and all patients signed a written informed consent form.

## Author Contributions

JW and CZ conceived and designed the study, as well as desiged the figures and tables. HD and HZ contributed to the statistical analysis, TW, ZR, YW, YC drafted and correcting the manuscript. All authors read and approved the final manuscript.

## Conflict of Interest

The authors declare that the research was conducted in the absence of any commercial or financial relationships that could be construed as a potential conflict of interest.

## Publisher’s Note

All claims expressed in this article are solely those of the authors and do not necessarily represent those of their affiliated organizations, or those of the publisher, the editors and the reviewers. Any product that may be evaluated in this article, or claim that may be made by its manufacturer, is not guaranteed or endorsed by the publisher.
